# Supplementary material for: Data-driven analysis and forecasting of highway traffic dynamics
Source: Nat Commun. 2020 Apr 29;11:2090. doi: 10.1038/s41467-020-15582-5 (PMC7190853; doi:10.1038/s41467-020-15582-5)
Supplement: Supplementary file 1 — Supplementary Information [file 41467_2020_15582_MOESM1_ESM.pdf]

Data-Driven Analysis and Forecasting of Highway Traffic Dynamics

Avila et al

Supplementary Figures

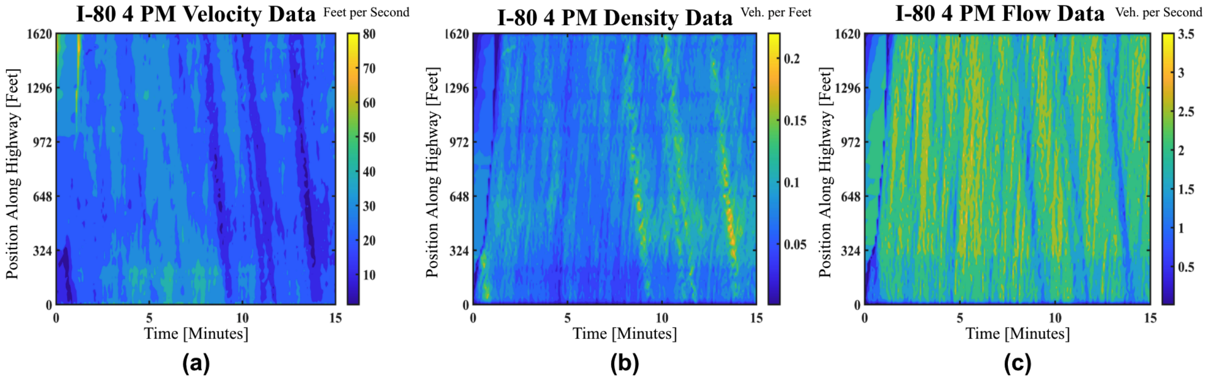

Supplementary Figure 1: US-80 4 pm spatiotemporal data sets. The data was collected between the hours of 4:00 pm to 4:15 pm. (1a) Velocity data set demonstrating the presence of moving localized cluster traffic jams. (1b) Density data set containing evidence of previously missing data. Specifically, a noticeable feature of low density is present in the far left of figure (1b) between the 300 feet and 1620 feet locations. (1c) Flow data also demonstrating the missing data feature in the far left region. The source data underlying figures 1a-d are provided in the Source Data file.

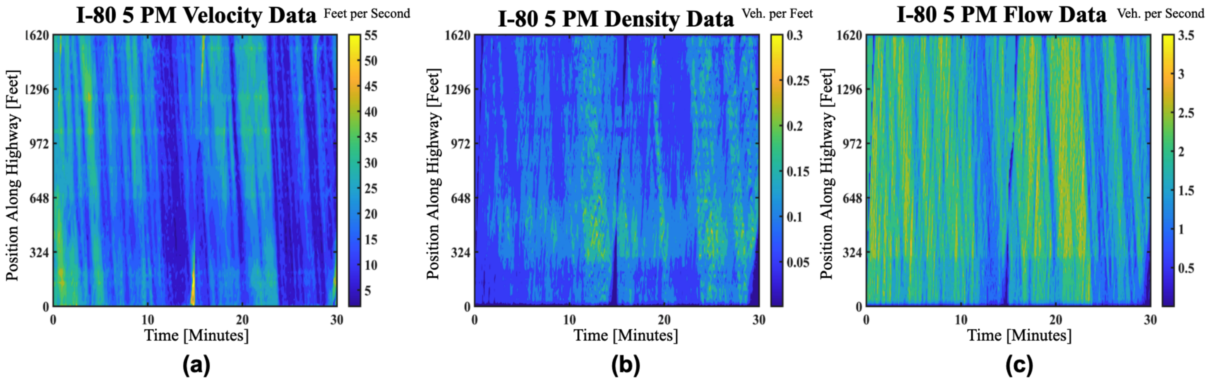

Supplementary Figure 2: US-80 5 PM Spatiotemporal Data Sets. The data was collected between the hours of 5:15 pm to 5:45 pm. (2a) Velocity data set demonstrating the presence of completely congested traffic.(2b) Density data set indicating that periods of slow moving traffic are a direct result of high density. (2c) Flow data demonstrating the missing data feature around the fifteen minute mark. The source data underlying figures 2a-d are provided in the Source Data file.

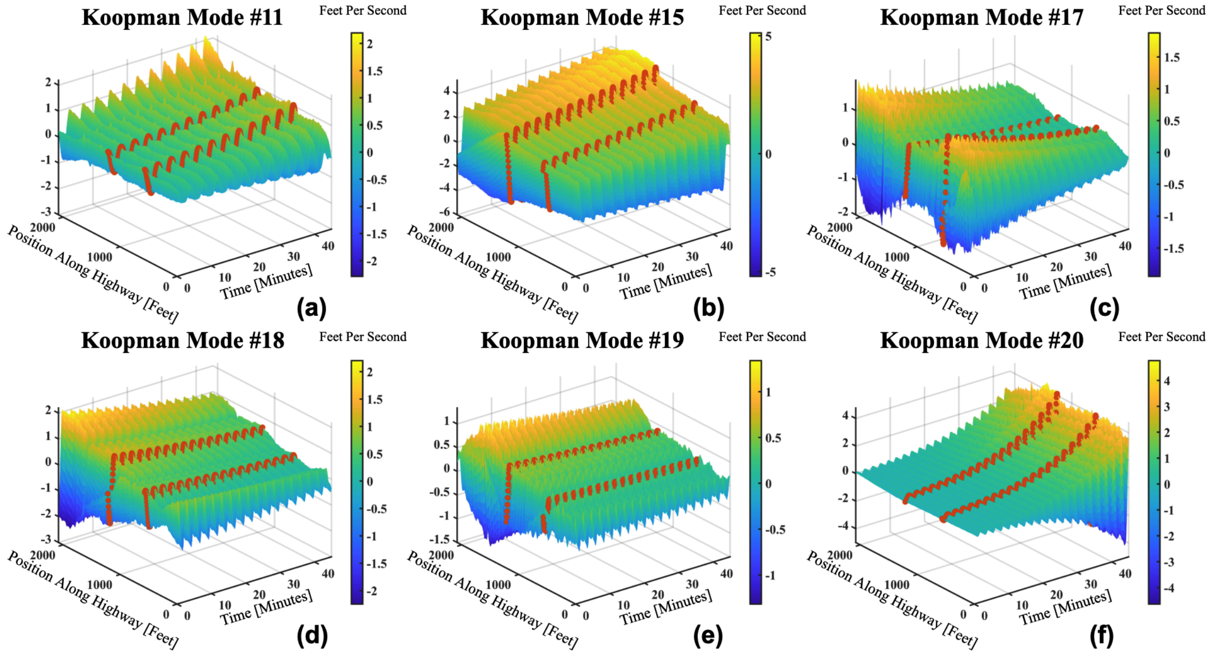

Supplementary Figure 3: Koopman modes for the US-101 velocity data demonstrating growing and decaying patterns. The dotted dark-orange line indicate the position of the on and off-ramps. Figures 3a, 3b, 3d and 3e all share the common structure of a pinned localized cluster (PLC). Specifically, their amplitude is entirely localized around the post-off-ramp (1280ft-2100ft) section of the highway. Figure 3c demonstrates a double-peaked structure. Lastly, figure 3f demonstrates a strong growth rate and is almost entirely active during the last fifteen minutes. A complete list containing the periods of oscillation of the modes we discussed can be referenced in supplementary table 1.

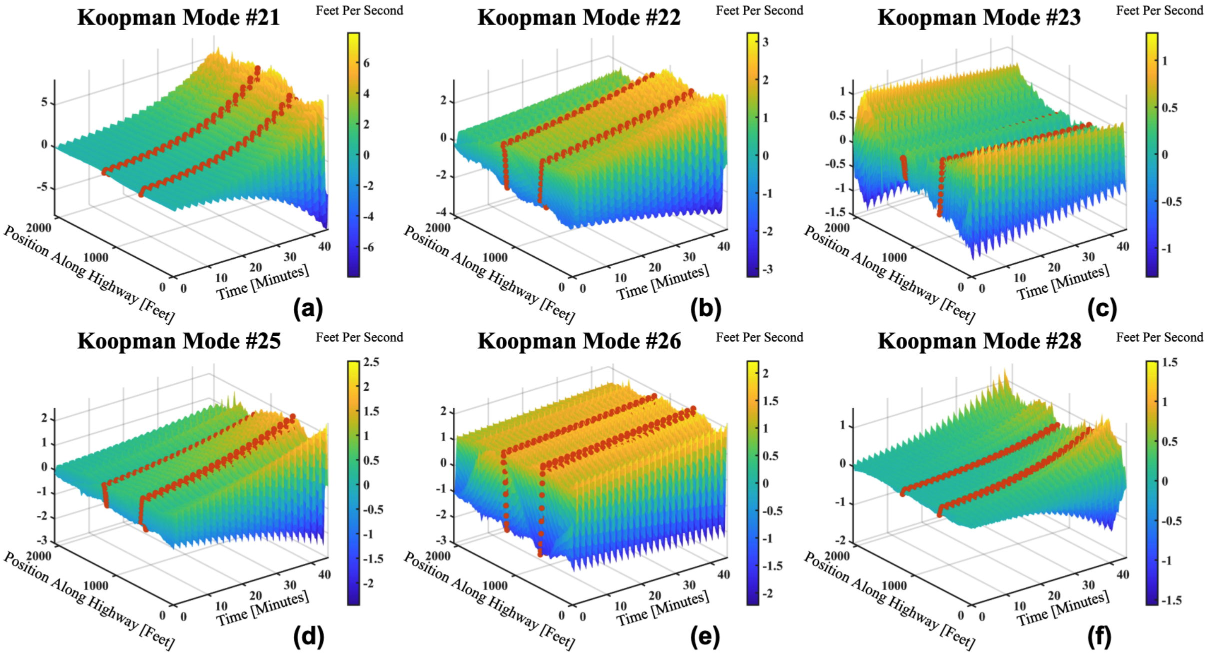

Supplementary Figure 4: Koopman modes for the US-101 velocity data demonstrating double peaked structures with node-like features. The dotted dark-orange lines indicate the position of the on and off-ramps. Figures 4a, 4b, 4d and 4f all demonstrate the ability of the KMD to identify growing patterns. Figure 4e corresponds to a highway wide traffic jam which propagates throughout the entire highway with an unperturbed amplitude. Lastly, figure 3c also possesses a double-peaked structure with its amplitude concentrated about the off and on-ramp locations. A complete list containing the periods of oscillation of the modes we discussed can be referenced in supplementary table 1.

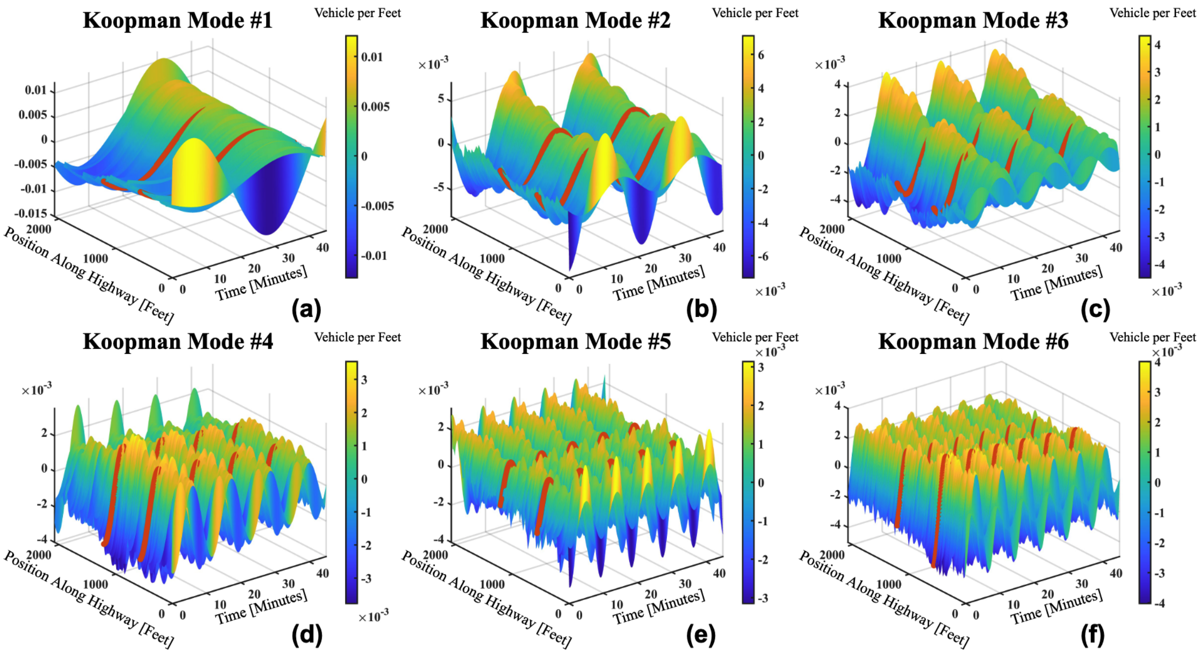

Supplementary Figure 5: The first six Koopman modes for the US 101 density data. The dotted dark-orange lines indicate the position of the on and off-ramps. Mode one in figure 5a corresponds to the initial transition of low to high-density traffic. It is also clear to see that modes two through four shown in figures 5b-5d are higher harmonics of the first mode. Mode five shown in figure 5e demonstrates the presence of peaked structures. Specifically, mode five has its largest amplitude within the post, mid and pre-ramp locations that are divided by nodes of near-zero amplitudes. Mode 6 shown in figure (5f) possess a large amplitude precisely over the on-ramp location of the highway and seems to capture the in-flowing traffic. A complete list containing the periods of oscillation of the modes we discussed can be referenced in supplementary table 1.

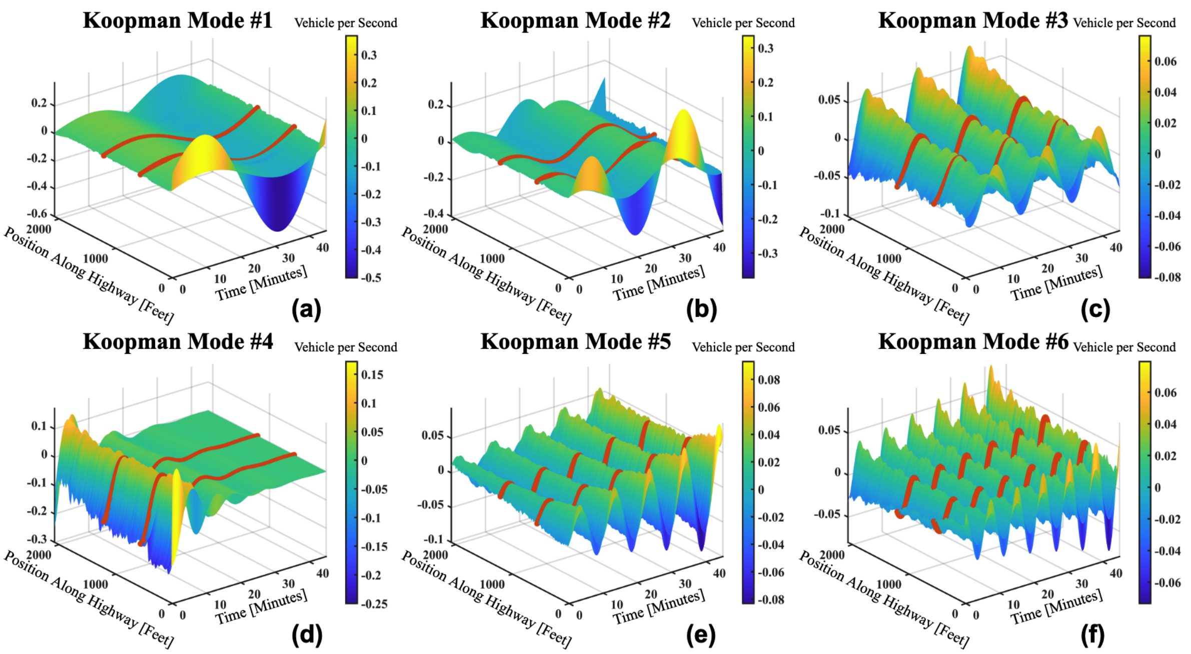

Supplementary Figure 6: The first six Koopman modes for the US 101 flow data. The dotted dark-orange lines indicate the position of the on and off-ramps. Modes one to three, shown in figures 6a-6c, are again harmonics of each other and represent the transition from free-flowing to congested traffic. Modes four and five, shown in figures 6d-6e, display prominent growing and decaying features. Mode six, shown in figure 6f, seems to correspond to waves of lower flow. This can be seen by observing that the majority of the amplitudes within mode six are negative with near-zero peaks. A complete list containing the periods of oscillation of the modes we discussed can be referenced in supplementary table 1.

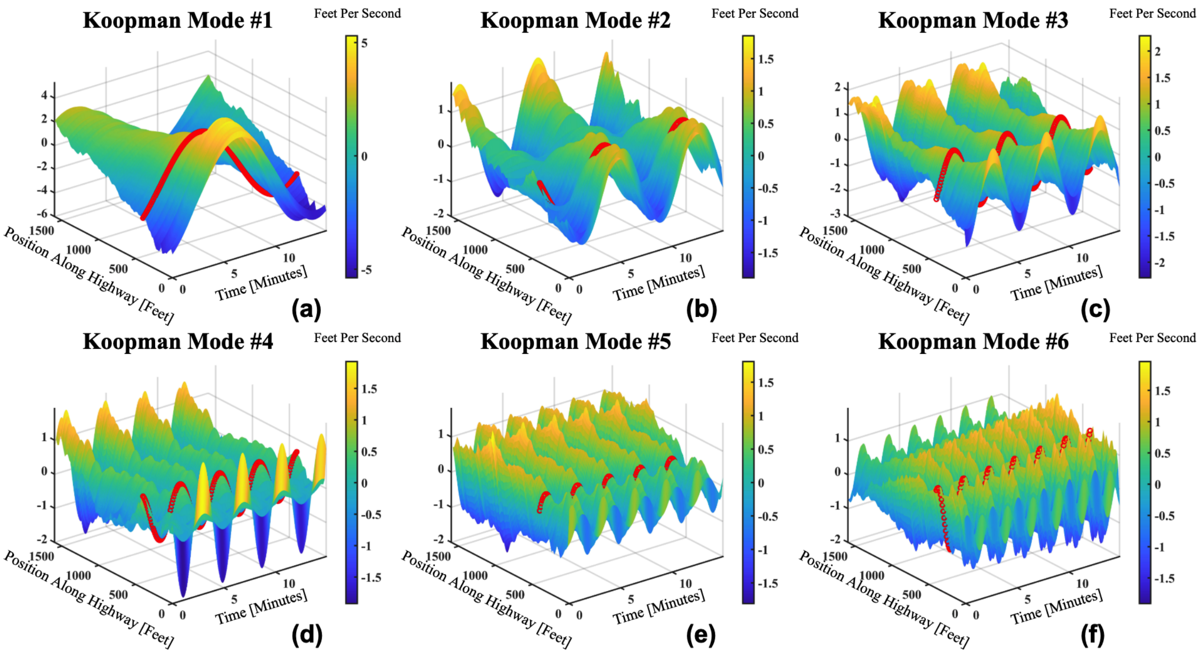

Supplementary Figure 7: The first six Koopman modes for the I-80 4 pm velocity data. The dotted dark-orange line indicates the position of the on-ramp. Mode one, shown in figure 7a, seems to be a pinned localized cluster (PLC) concentrated on the on-ramp section of the highway. It also seems to capture the transition from high to low velocities. Modes two through five, shown in figure 7b-7e, also seem to display a PLC structure and correspond to harmonics of the first mode. However, they also have a peak in amplitude near the end of the highway. Mode six, shown in figure 7f, provides further evidence for the pumping effect in that a noticeable increase followed by a decrease in amplitude occurs as the wave propagates along the highway. A complete list containing the periods of oscillation of the modes we discussed can be referenced in supplementary table 1.

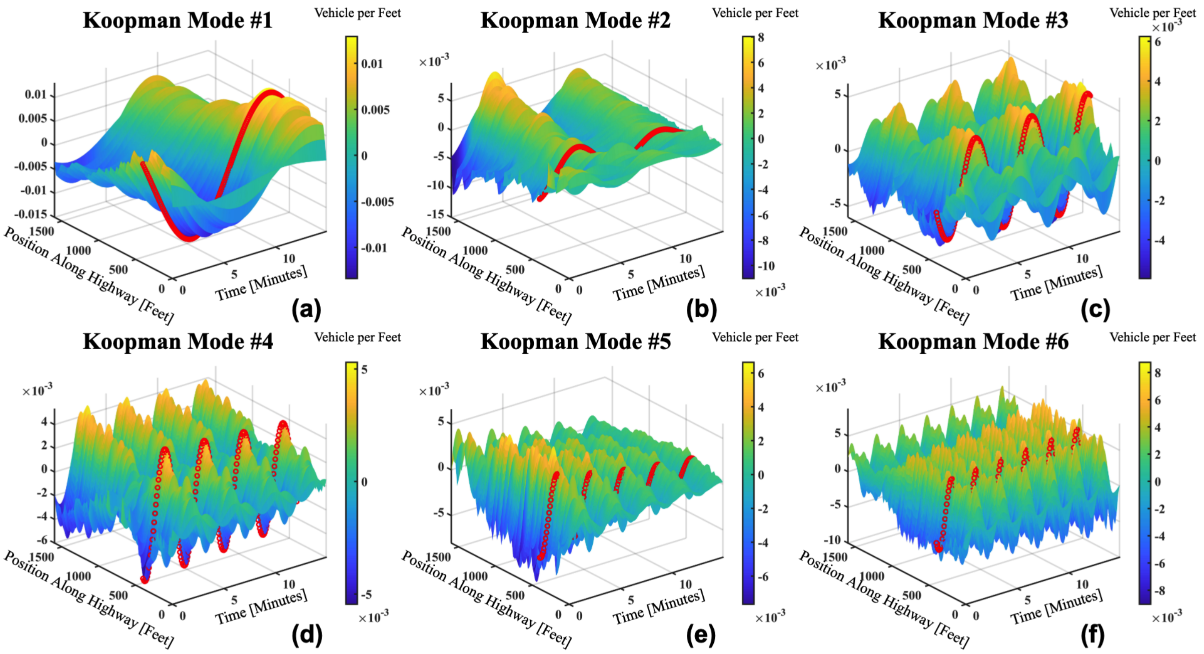

Supplementary Figure 8: The first six Koopman modes for the I-80 4 pm density data. The dotted dark-orange line indicates the position of the on-ramp. As one would expect the first mode, shown in figure 8a, seems to be an exact inverse of figure 7a. This first mode also seems to be capturing the transition from low to high densities that occurs. Modes two through four, shown in figures 8b-8d, are also harmonics of the first, however, modes three and four seem to also display a peak in amplitude near the on-ramp section of the highway. Modes five and six, shown in figures 8e-8f, display a drop in amplitudes as they travel along the highway. A complete list containing the periods of oscillation of the modes we discussed can be referenced in supplementary table 1.

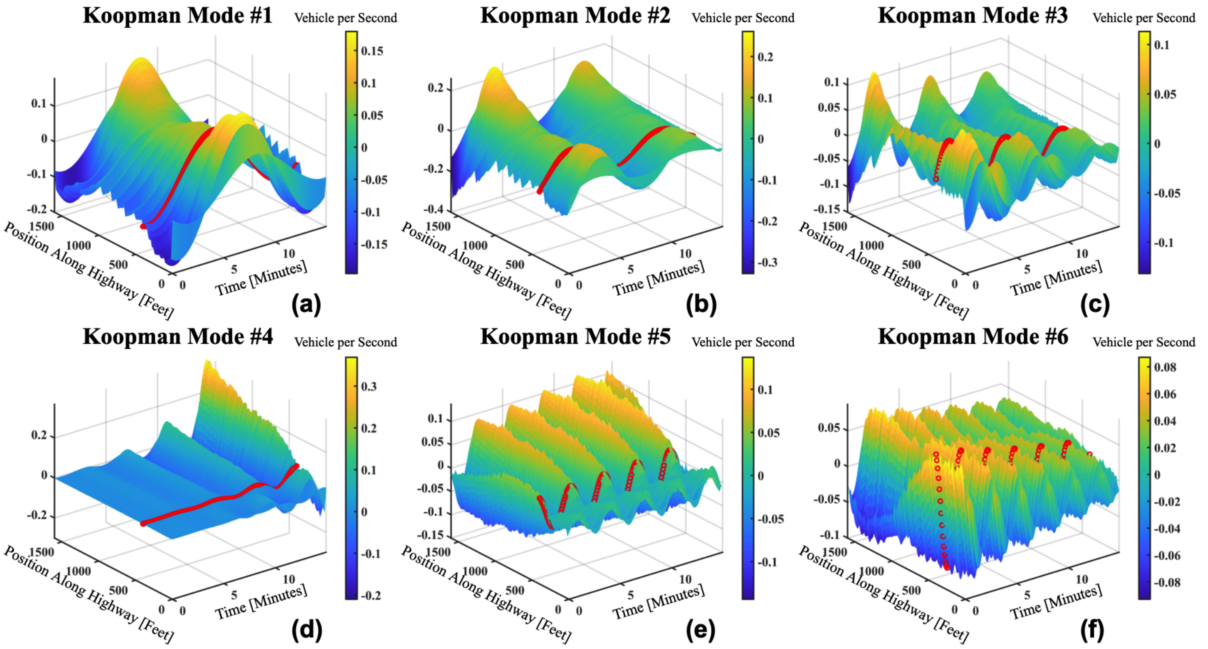

Supplementary Figure 9: The first six Koopman modes for the I-80 4 pm flow data. The dotted dark-orange line indicates the position of the on-ramp. Modes one to three, shown in figures 9a-9c are harmonics of each other and also correspond to the transition of high to low flow. Mode five, shown in figure 9e seems to be entirely concentrated in the post-on-ramp section of the highway. Furthermore, the structure of mode five strongly resembles a stop and go wave pattern. Mode six, shown in figure 9f, seems to also display a double-peaked structure concentrated over the on-ramp and post-on-ramp sections. Lastly, mode four, shown in figure 9d exhibits a very strong growth rate. Specifically, the amplitude of mode four is nearly zero for the majority of the fifteen minutes. A complete list containing the periods of oscillation of the modes we discussed can be referenced in supplementary table 1.

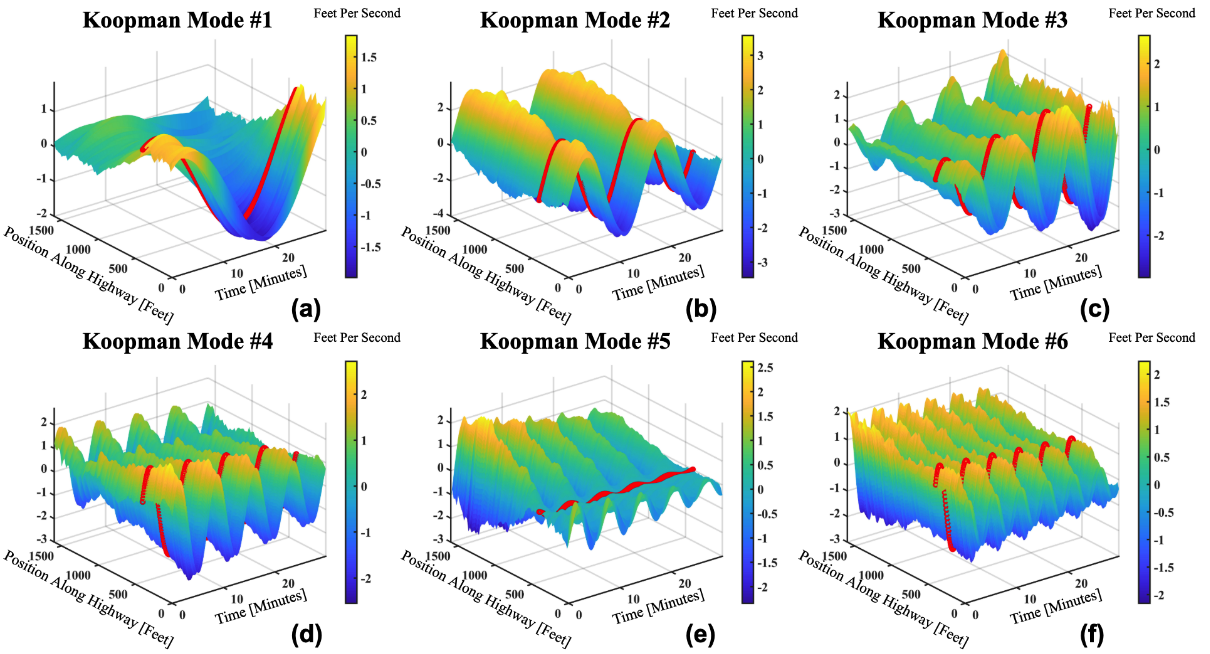

Supplementary Figure 10: First six Koopman modes for the I-80 5 pm velocity. The dotted dark-orange line indicates the position of the on-ramp. Modes one, three and four, shown in figures 10a, 10c-10d, seem to correspond to pinned localized clusters (PLC) concentrated over the on-ramp. They also seem to capture the general transition from high to low velocities. Mode two, shown in figure 10b, corresponds to a moving localized cluster (MLC) traffic jam. Mode five, shown in figure 10e, exhibits seems to decay in time and resembles a PLC that is concentrated about the post-on-ramp section of the highway. Similar to mode two, mode six, shown in figure 10f, also corresponds to an MLC that propagates along the entire highway. A complete list containing the periods of oscillation of the modes we discussed can be referenced in supplementary table 1.

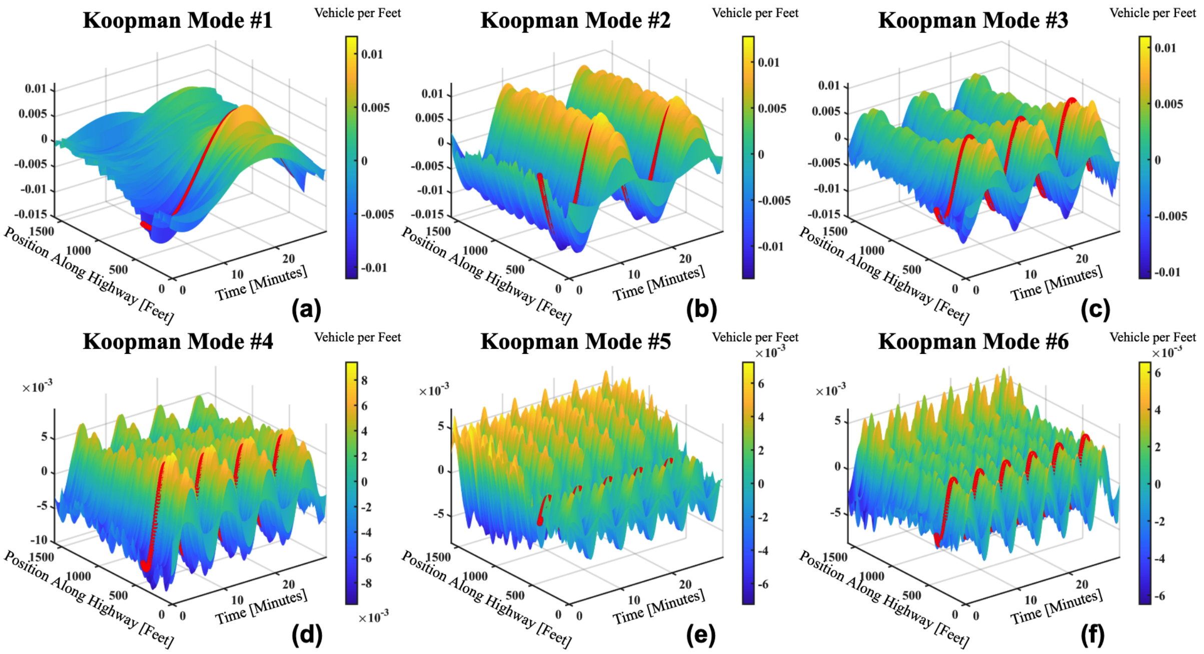

Supplementary Figure 11: The first six Koopman modes for the I-80 5 pm density. The dotted dark-orange line indicates the position of the on-ramp. Modes one, three, and four, shown in figures 11a, 11c-11d, are pinned localized clusters (PLC) that are concentrated about the on-ramp section of the highway. Mode two, shown in figure 11b, corresponds to a moving localized cluster (MLC) traffic jam. The traffic jam seems to propagate and affect the entire highway and carries a noticeable peak in amplitude over the on-ramp section of the highway. Modes five and six, shown in figures 11e-11f, seem to be PLC's that are concentrated about the post-on-ramp section of the highway. It is also interesting to note that mode five possesses a node of near-zero amplitude at precisely the on-ramp location. A complete list containing the periods of oscillation of the modes we discussed can be referenced in supplementary table 1.

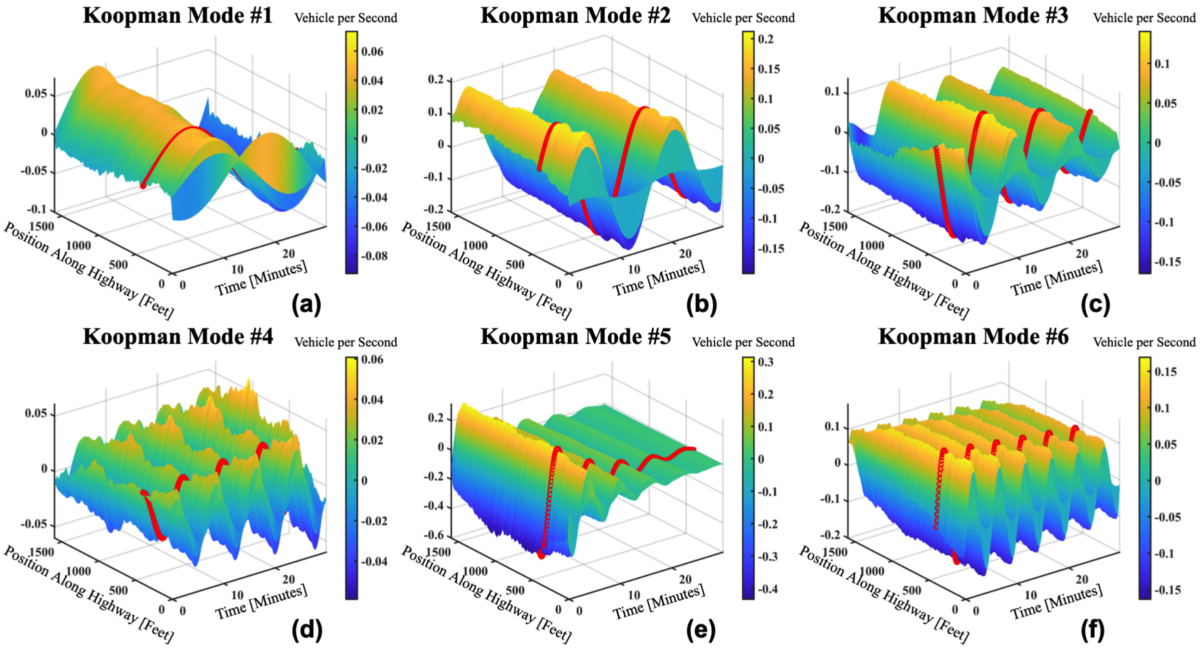

Supplementary Figure 12: The first six Koopman modes for the I-80 5 pm flow. The dotted dark-orange line indicates the position of the on-ramp. Modes one to three and six, shown in figures 12a-12c and 12f, clearly display the features of a moving localized cluster (MLC) traffic jam. Specifically, they propagate across the entire highway without much perturbation to their amplitudes. Mode five, shown in figure 12e also corresponds to an MLC jam, however, it also possesses a strongly decaying feature. Specifically, it is only active for approximately, the first ten minutes. Lastly, mode four, shown in figure 12d seems to possess peaks in amplitude at three distinct locations throughout the highway. This further demonstrates the existence of patterns with peaked structures that have concentrated amplitude in certain regions of the highway. A complete list containing the periods of oscillation of the modes we discussed can be referenced in supplementary table 1.

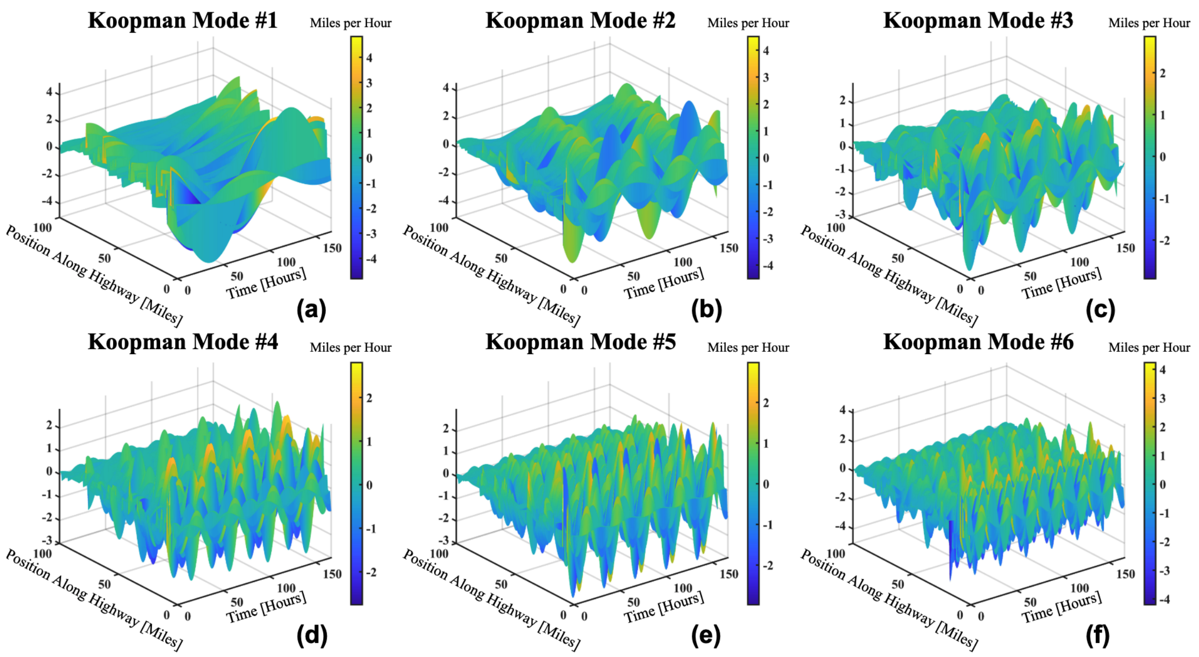

Supplementary Figure 13: First six Koopman Modes for the I-10 highway velocity data demonstrating intraweek Patterns. The first mode, shown in figure 13a, corresponds to a period of roughly seven days. Interestingly, the positive amplitudes within mode one correspond to the Friday through Monday period while negative amplitudes occur during the middle of the week. This indicates that traffic is much heavier on Fridays and Mondays as opposed to the midweek. Furthermore, modes two through six, shown in figures 13b-13f, correspond to higher harmonics of the weekly mode and capture the biweekly, triweekly and intraweekly patterns. These intraweekly patterns seem to be highly periodic with very low growth or decay rates. Overall, patterns corresponding to periods of seven days down to fourteen hours are identified within the first twelve Koopman modes associated with the weekly I-10 data set. A complete list containing the periods of oscillation of the modes we discussed can be referenced in supplementary table 1.

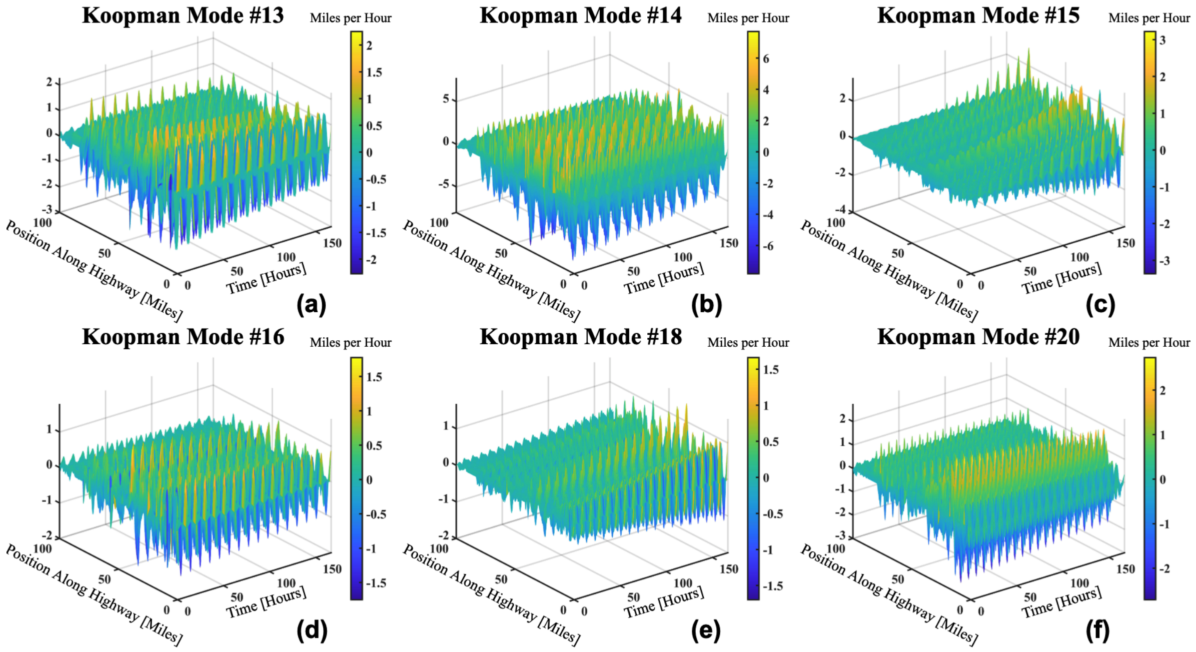

Supplementary Figure 14: Higher Koopman modes for the I-10 highway velocity data demonstrating growing and decaying patterns. An interesting feature of these modes is that despite being intraday modes some seem to be slightly more active on the weekends as opposed to the week. Specifically, modes fifteen and eighteen, shown in figures 14c and 14e, display a noticeable increase in amplitude toward the end of the week. On the other hand, modes thirteen, fourteen, sixteen and twenty, shown in figures 14a-14b, 14d and 14f, display slight decays in their amplitude during the week. This indicates that although all of these patterns are active throughout every day of the week some exhibit themselves more strongly during the week or weekend. A complete list containing the periods of oscillation of the modes we discussed can be referenced in supplementary table 1.

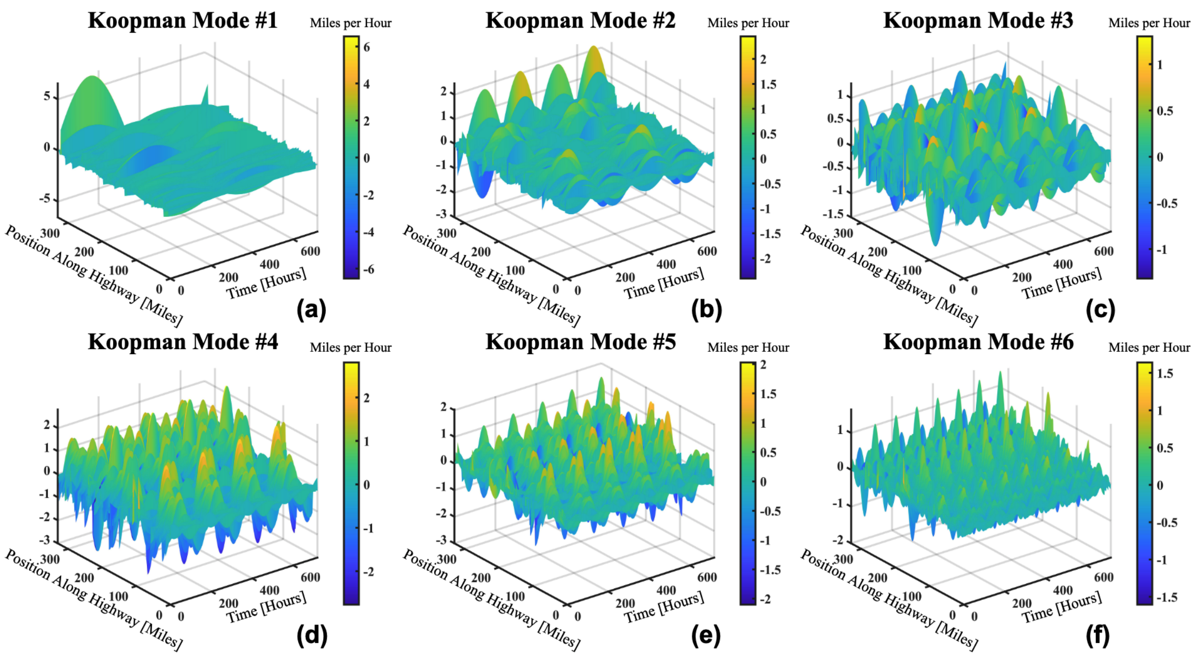

Supplementary Figure 15: The first six modes for the I-5 highway demonstrating intramonthly patterns. (15a) The first mode corresponds to the monthly pattern and demonstrates very low amplitudes indicating that traffic is roughly regular on a monthly scale. (15b-f) Higher harmonics of mode one corresponding to the bimonthly and intramonthly patterns and appear to be rather periodic with little growth or decay. Some of the largest amplitudes are near the one hundred and the fifty-mile section of the highway corresponding to the city of Los Angeles, which is known to have the heaviest traffic among the cities covered by this section of the I-5 highway. A complete list containing the periods of oscillation of the modes we discussed can be referenced in supplementary table 1.

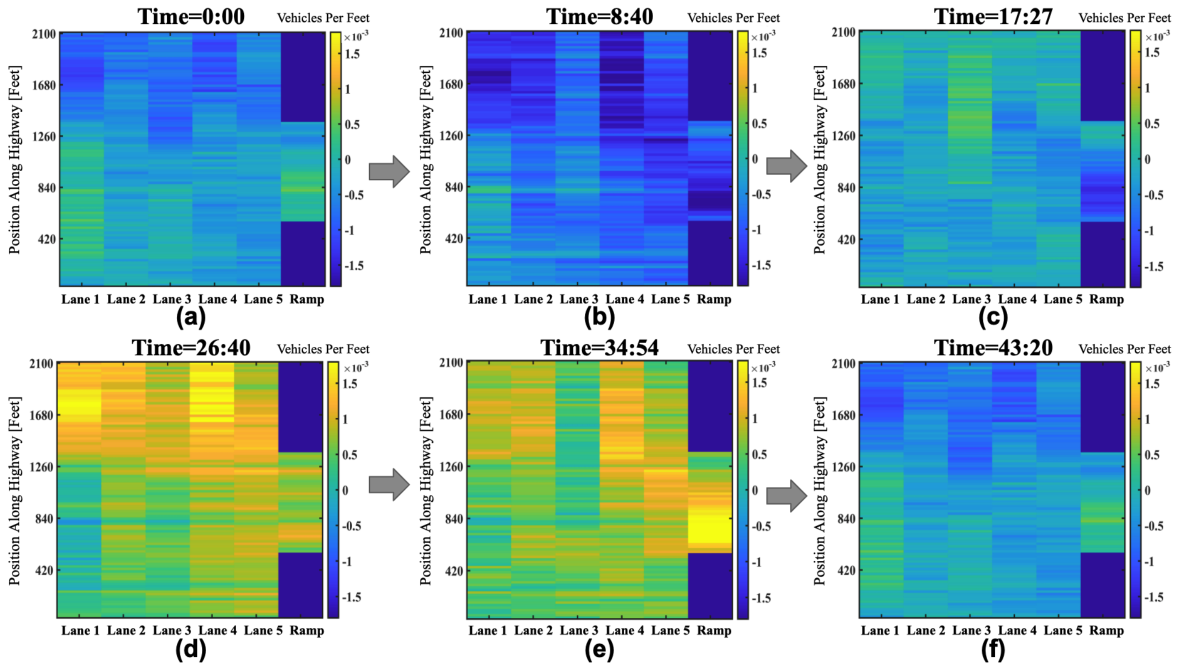

Supplementary Figure 16: Video snapshots of the first multi-lane mode demonstrating multi-lane pinned localized cluster patterns. The time for each figure is given in minutes-seconds and the on-ramp densities have been multiplied by five for visual purposes. The first multi-lane Koopman mode demonstrates a noticeable concentration of vehicle densities in the post-off-ramp section of the highway and carries the features of a multi-lane pinned localized cluster (PLC) structure. This is in agreement with the first mode of the corridor-wide analysis and the fact that PLC's are typically found near bottlenecks such as on and off-ramps. Furthermore, it is clear to see that not all the lanes are affected the same. We remind and strongly encourage the reader to also reference supplementary video 1, available in the online supplementary information. A complete list of the periods for the multi-lane modes can be referenced in supplementary table 1.

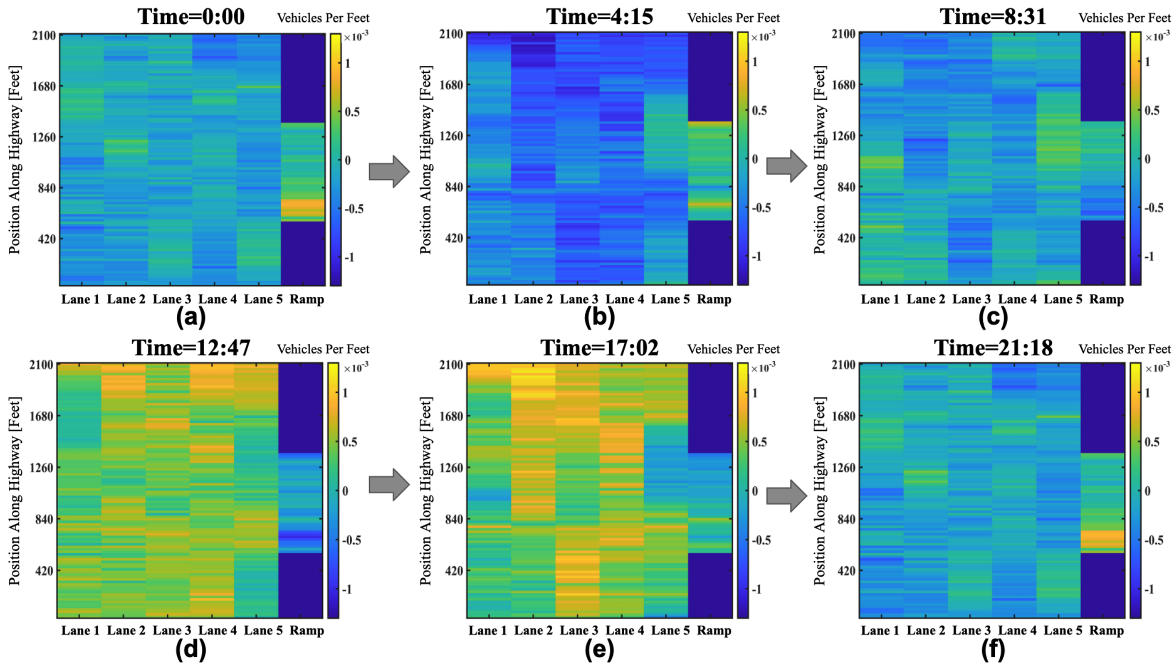

Supplementary Figure 17: Video snapshots of the second multi-lane mode. The time for each figure is given in minutes-seconds and the on-ramp densities have been multiplied by five for visual purposes. The second mode corresponds to a half period harmonic of the first and also resembles a multi-lane pinned localized structure (PLC). The interesting feature to note is how the on-ramp densities are clearly out of phase with the highway densities. Indicating that the vehicles entering the highway enter when there is currently no congestion on the highway. This confirms that the ramp is properly metered on the timescale of this mode. We remind and strongly encourage the reader to also reference supplementary video 2, available in the online supplementary information. A complete list of the periods for the multi-lane modes can be referenced in supplementary table 1.

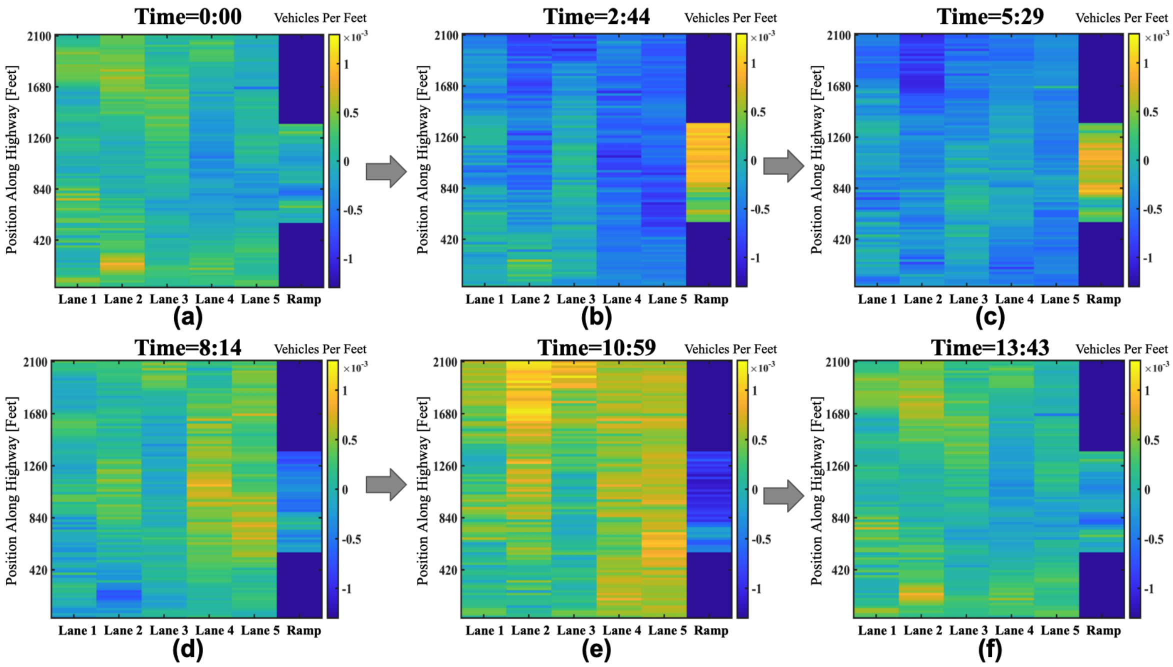

Supplementary Figure 18: Video snapshots of the third multi-lane mode. The time for each figure is given in minutes-seconds and the on-ramp densities have been multiplied by five for visual purposes. This mode also corresponds to a harmonic of the first and noticeably displays the successful ramp-metering of vehicles. Figure 18b and 18e clearly display how the on-ramp traffic is low precisely when the highway traffic is high and vice-versa. We remind and strongly encourage the reader to also reference supplementary video 3, available in the online supplementary information. A complete list of the periods for the multi-lane modes can be referenced in supplementary table 1.

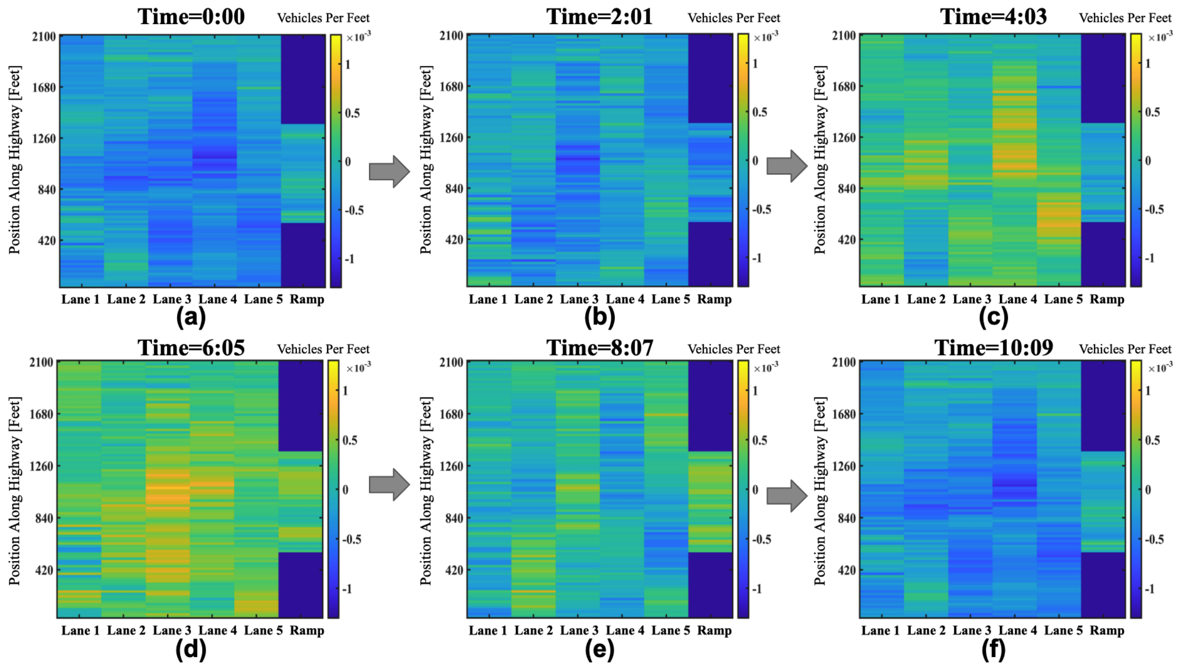

Supplementary Figure 19: Video snapshots of the fourth multi-lane mode. The time for each figure is given in minutes-seconds and the on-ramp densities have been multiplied by five for visual purposes. The fourth mode begins to display the lane changing maneuvers that can occur within the mid-ramp section of the highway. Specifically, figure 19c displays the onset of congestion occurs within lane four and five. However, proceeding to figure 19d one can clearly see how the congestion travels diagonally into lane three and figure 19e displays how the congestion travels as far as lane two. We remind and strongly encourage the reader to also reference supplementary video 4, available in the online supplementary information. A complete list of the periods for the multi-lane modes can be referenced in supplementary table 1.

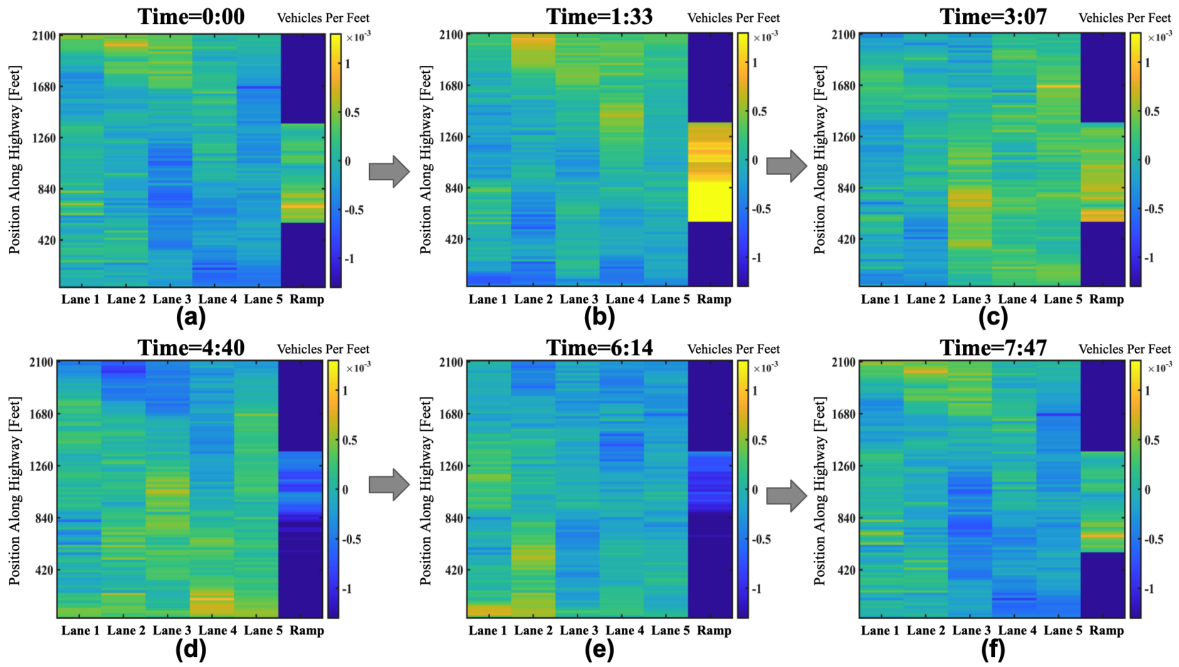

Supplementary Figure 20: Video snapshots of the fifth multi-lane mode. The time for each figure is given in minutes-seconds and the on-ramp densities have been multiplied by five for visual purposes. Starting with figure 20b one can see how the increase in merging traffic begins to cause congestion within lanes five and four. Proceeding to figures 20c and 20d one can see the congestion propagates laterally over to lane three and slightly into lane two. Finally, figure 20e demonstrate how the congestion eventually propagates into lanes one and two before traveling out of the region of study. The fifth mode displays how the merging of incoming vehicles can be a catalyst for lateral traffic waves. We remind and strongly encourage the reader to also reference supplementary video 5, available in the online supplementary information. A complete list of the periods for the multi-lane modes can be referenced in supplementary table 1.

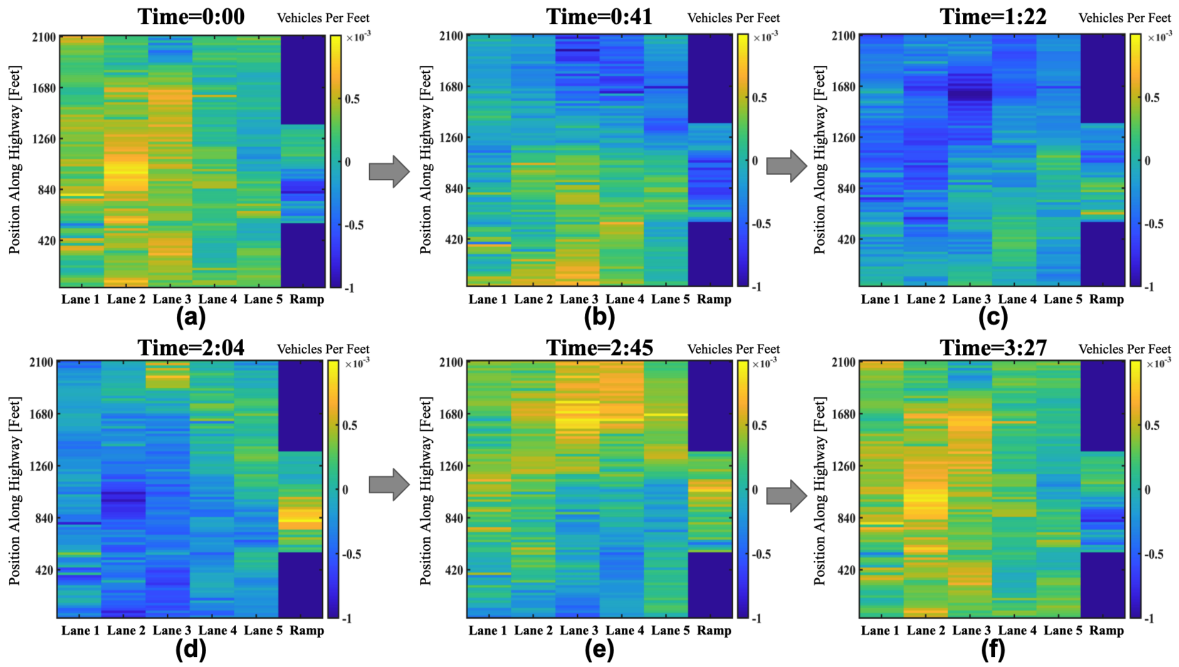

Supplementary Figure 21: Video snapshots of the tenth multi-lane mode. The time for each figure is given in minutes-seconds and the on-ramp densities have been multiplied by five for visual purposes. Mode ten displays a prominent zig-zag lane changing behavior that can be seen by first observing the congestion within lanes three, four and five within figure 21e. Then proceeding to figures 21f and 21a one can clearly see the lateral propagation of the congestion into lanes one, two and three. However, proceeding to figures 21b one can observe that the congestion has propagated back into lanes three, four and five. It is evident that mode ten displays an overall zig-zag motion that begins in lanes three, four and five propagates laterally to lanes one, two and three and then returns to lanes three, four and five before traveling beyond the area of study. The extraction of such zig-zag patterns from raw data alone is again a novel pattern identified by the Koopman mode decomposition. We remind and strongly encourage the reader to also reference supplementary video 10, available in the online supplementary information. A complete list of the periods for the multi-lane modes can be referenced in supplementary table 1.

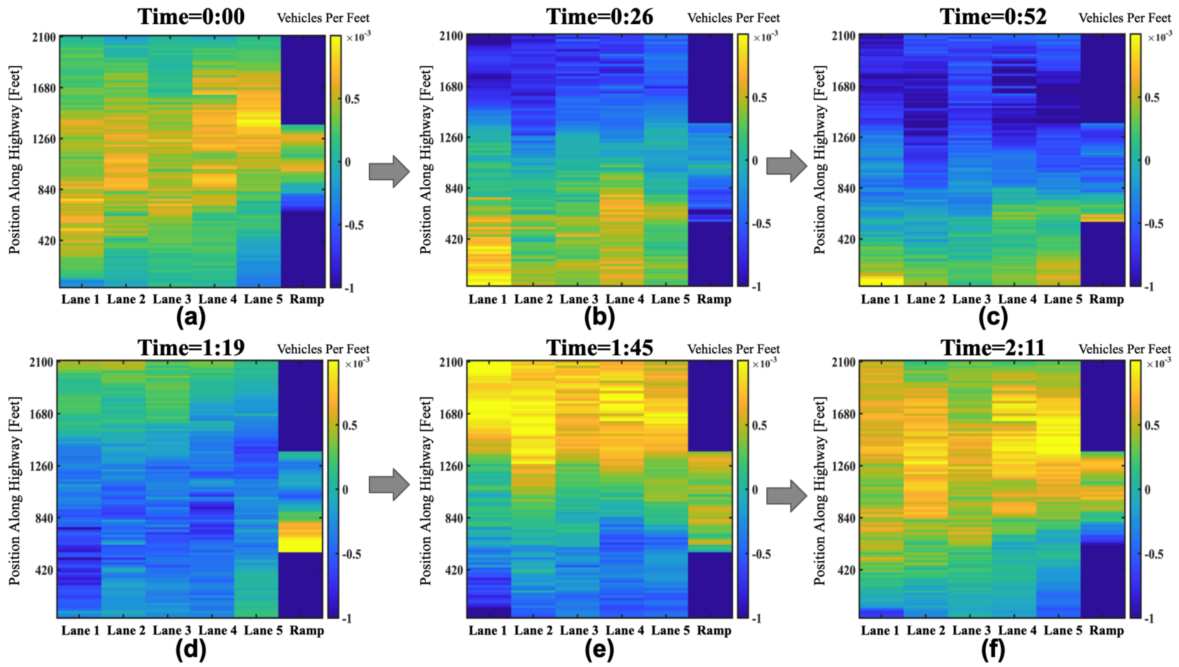

Supplementary Figure 22: Video snapshots of the fourteenth multi-lane mode. The time for each figure is given in minutes-seconds and the on-ramp densities have been multiplied by five for visual purposes. Mode fourteen is a clear harmonic of mode seven discussed within the main text. By observing the sequence of figures starting from figure 22d, 22e, 22f and proceeding to figures 22a, 22b and 22c one can see that mode fourteen corresponds to a multi-lane moving localized cluster (MLC). This highway wide traveling traffic jam is unfortunately in phase with the merging traffic. This can be seen by observing figures 22e, 22f and 22a and noticing that incoming traffic is merging precisely as the highway jam is propagating by. We remind and strongly encourage the reader to also reference supplementary video 14, available in the online supplementary information. A complete list of the periods for the multi-lane modes can be referenced in supplementary table 1.

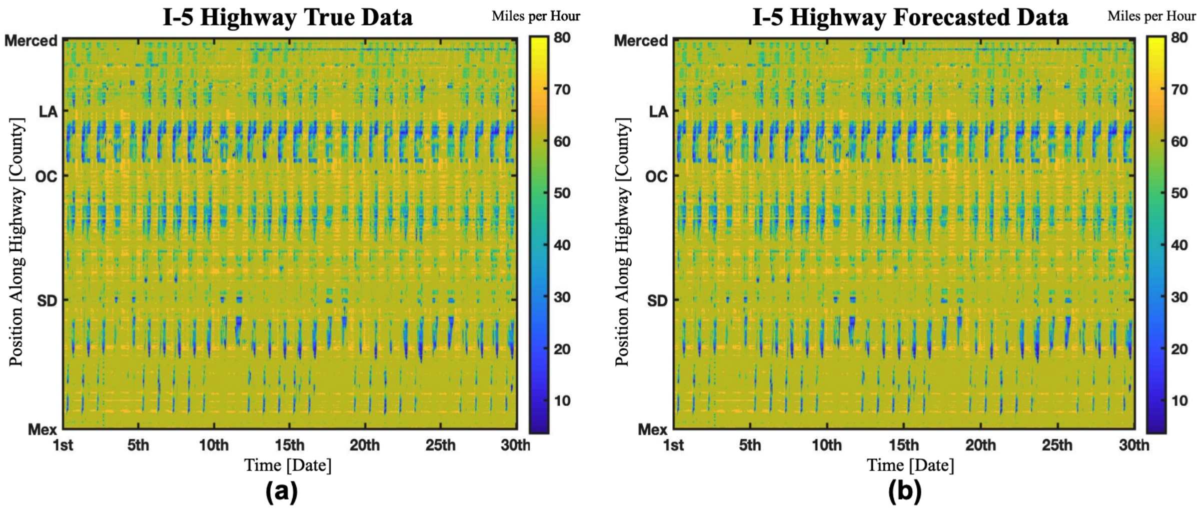

Supplementary Figure 23: Comparison of true and forecasted data for the northbound I-5 highway. The high similarity between the data sets confirms that the performance of the MH-HDMD algorithm is stable even for timescales on the order of a month. The source data underlying figures 23a are provided in the Source Data file.

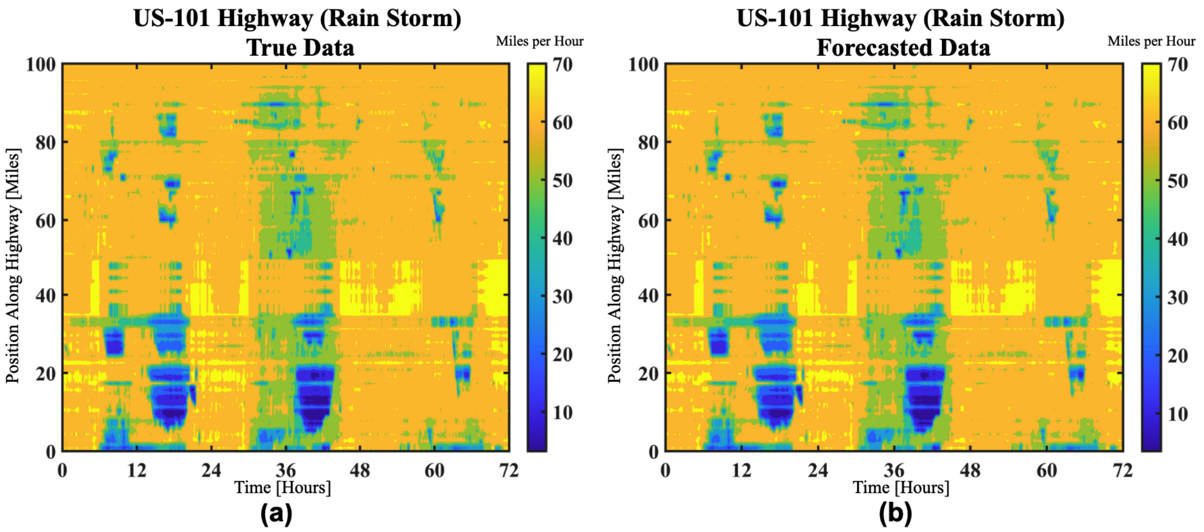

Supplementary Figure 24: Comparison of true and forecasted data for the US-101 highway. The data was collected for the day before during and after the deadly southern California rainstorm[1] that occurred on February 17 2017. The high similarity of the data sets confirms that the performance of the MH-HDMD algorithm is stable even under adverse weather conditioned traffic. The source data underlying figures 24a are provided in the Source Data file.

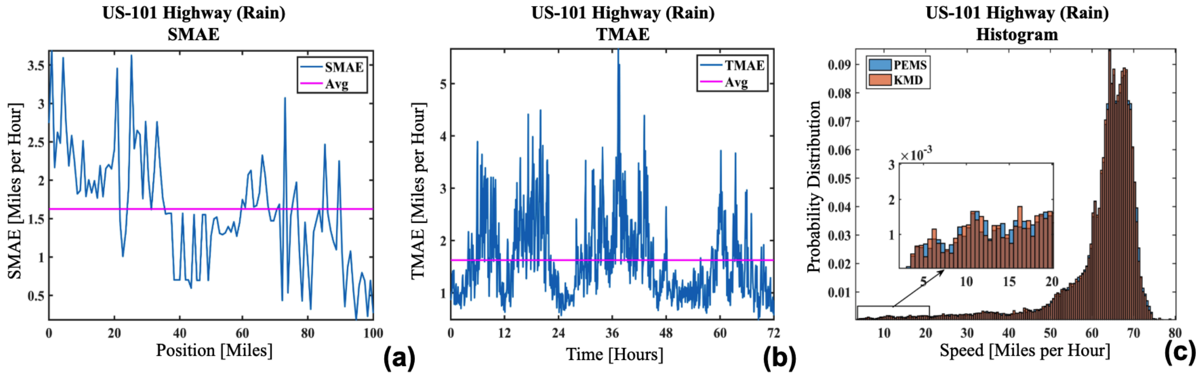

Supplementary Figure 25: Error analysis for the US-101 rainstorm data set. Figures 25a and 25b confirm that our spatial and temporal mean absolute errors are on average between one to two miles per hour. Figure 25c confirms that the statistics of our forecasted data closely matches the statistics of the raw data. Overall, these figures confirm that our forecasts are robust even under adverse weather conditioned traffic.

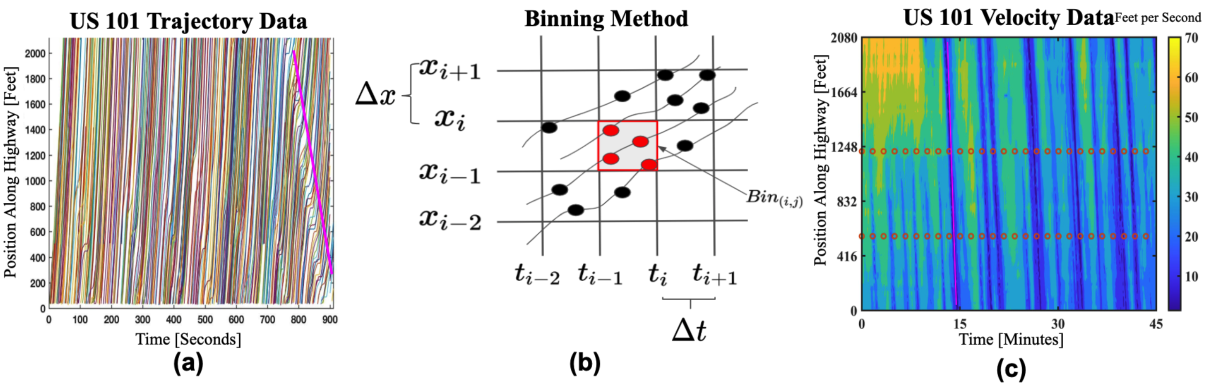

Supplementary Figure 26: Flow scheme for obtaining spatiotemporal data from NGSIM trajectory data. (26a) trajectory data from the first fifteen minutes of video. Each colored line corresponds to an individual vehicle. We have highlighted the traffic jam present within the trajectory data with a magenta-colored line. (26b) Schematic of the binning method utilized to generate our spatiotemporal data. The spatiotemporal domain is divided into bins in which the quantities of interest (velocity, density, and flow) are computed according to the formulas described within the methods section. (26c) Spatiotemporal plot of the velocity profile for the US-101 highway. It is clear to see how the magenta-colored traffic jam from figure 26a is also present within the spatiotemporal data. This indicates that the binning method preserves the dynamic structures occurring within the trajectory data. The dark orange dotted lines correspond to the locations of the on and off-ramps.

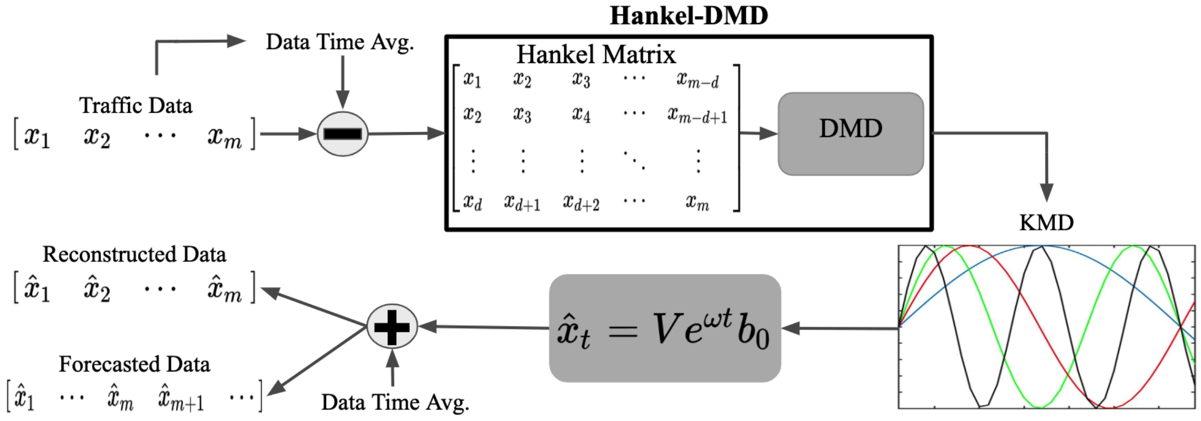

Supplementary Figure 27: Flow scheme demonstrating how Hankel-DMD on mean subtracted traffic data enables the computation of spatiotemporal patterns, reconstructions, and forecasts. First, the time average of the traffic data matrix is removed. Then the spectral quantities (eigenvalues, modes) of the Koopman operator associated with the mean subtracted data are computed via the Hankel-DMD algorithm. Hankel-DMD consists of applying an exact DMD algorithm [2] to a time-delay embedded data matrix (Hankel matrix). The dimension of the embedded Hankel matrix is now $d \cdot n$ by $m - d$, where d is the number of delays and n is the size of a single data vector $\mathbf{x}_\mathrm{i}$. Hence a taller data matrix (more rows) is obtained, at the expense of losing d columns. The modes of the Koopman operator can then be evolved via the linear equation they satisfy[3,4] and superimposed with the previously removed time averages to obtain a reconstruction of the original traffic data or produce forecasts. Pseudocode of the Hankel-DMD algorithm can be referenced in supplementary note 1 and the corresponding source code is made available according to the code availability statement in the main text.

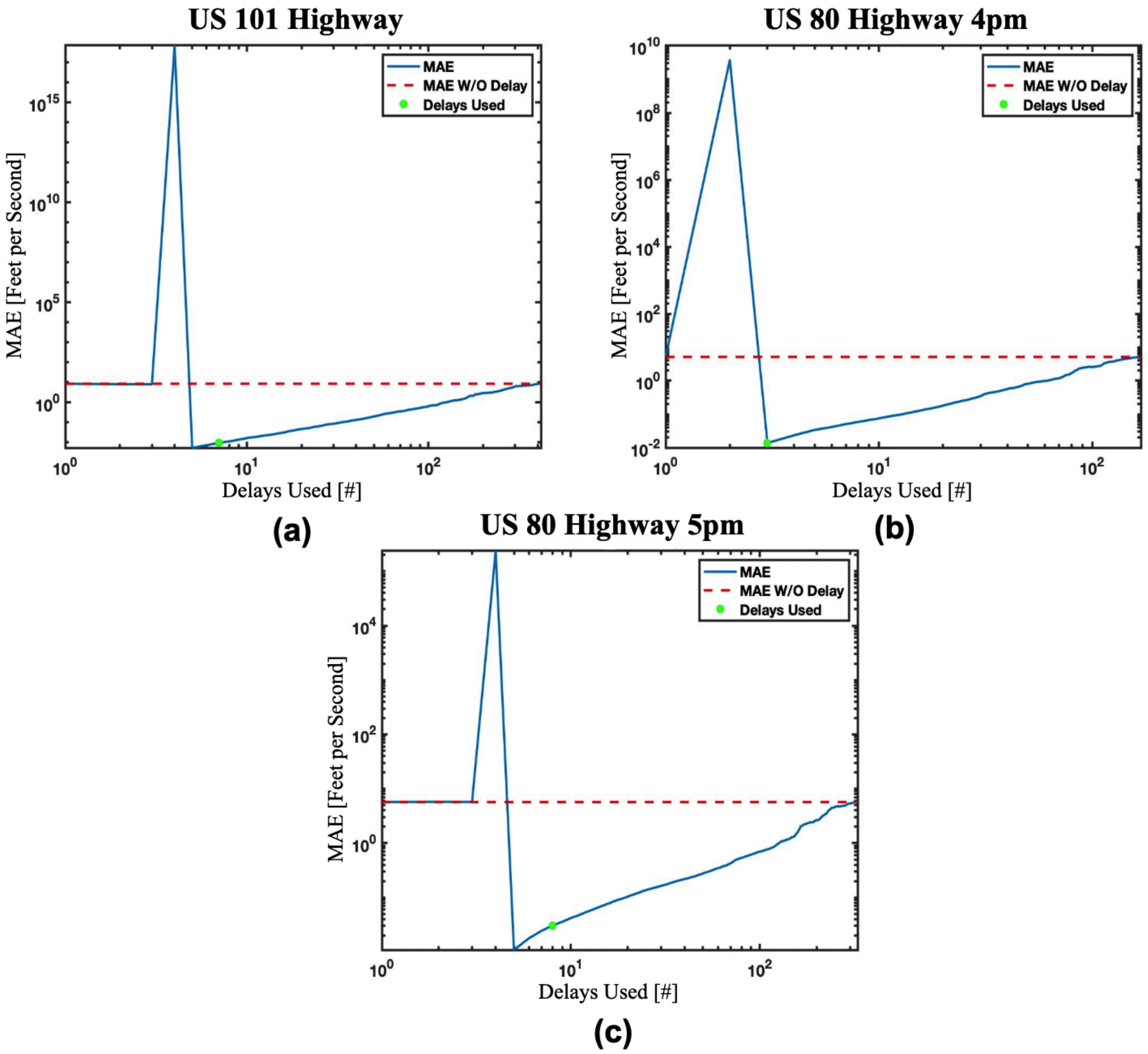

Supplementary Figure 28: Log-log plot of the reconstruction mean absolute error. Figures 28a-c clearly verify our reasoning for choosing enough delays such that the embedded matrix is at least tall. Interestingly, every case demonstrates several orders of magnitude in reconstruction error when the matrices are near square but still fat. Beyond the square threshold, the MAE drops and takes an entire order of magnitude in delays before returning to its original value (dashed red curve). The green dot represents the number of delays we used in our analysis and were determined according to the systematic procedure outlined in the methods section. The plots confirm that our choice for the delay was always near-optimal in every scenario.

Mode [#]	US-101 Velocity	US-101 Density	US-101 Flow	US-101 Multi-lane	I-80 4pm Velocity	I-80 4pm Density	I-80 4pm Flow	I-80 5pm Velocity	I-80 5pm Density	I-80 5pm Flow	I-10 Velocity	I-5 Velocity	Multi-lane Network
1	44.20	45.96	45.92	46.19	15.08	14.85	12.94	30.89	30.48	30.70	167.03	720.40	24.00
2	22.03	22.12	26.96	23.06	7.59	7.99	8.66	14.96	14.78	17.06	83.41	358.37	11.99
3	15.71	15.85	14.97	15.44	5.18	5.42	5.43	9.82	9.97	11.04	55.73	239.60	8.00
4	11.45	11.34	11.93	11.56	3.82	3.82	3.91	7.40	7.58	7.76	41.51	179.94	6.00
5	9.19	9.05	10.21	9.25	3.05	3.24	3.21	6.33	5.91	6.11	33.70	143.61	4.81
6	7.55	7.58	7.68	7.72	2.48	2.44	2.59	5.13	5.15	5.10	27.81	119.82	4.02
7	6.44	6.55	6.75	6.61	2.18	2.22	2.12	4.37	4.30	4.28	23.89	101.92	3.44
8	5.61	5.62	5.61	5.78	1.91	1.93	1.97	3.64	3.73	3.57	21.04	91.05	3.02
9	4.91	4.97	5.17	5.13	1.63	1.64	1.65	3.34	3.31	3.42	18.56	80.17	2.68
10	4.37	4.38	4.49	4.62	1.43	1.43	1.43	3.00	3.02	2.91	16.78	71.92	2.41
11	3.94	4.10	4.06	4.19	1.40	1.34	1.35	2.70	2.68	2.72	15.18	65.66	2.19
12	3.56	3.70	3.98	3.84	1.24	1.30	1.23	2.52	2.51	2.44	14.04	60.33	2.01
13	3.47	3.38	3.50	3.54	1.18	1.17	1.19	2.29	2.28	2.37	12.83	55.43	1.85
14	3.30	3.14	3.29	3.29	1.08	1.06	1.06	2.14	2.12	2.17	11.93	52.02	1.72
15	3.11	3.09	2.98	3.08	1.00	0.99	0.98	2.00	1.99	2.02	11.12	47.55	1.60
16	2.89	2.85	2.89	2.89	0.94	0.95	0.90	1.89	1.86	1.90	10.50	45.48	1.50
17	2.69	2.65	2.65	2.71	0.89	0.88	0.85	1.74	1.74	1.69	9.88	42.45	1.41
18	2.53	2.43	2.52	2.58	0.83	0.81	0.84	1.62	1.61	1.66	9.31	40.15	1.33
19	2.38	2.42	2.37	2.44	0.78	0.79	0.78	1.55	1.60	1.56	8.88	37.88	1.26
20	2.26	2.29	2.22	2.31	0.75	0.74	0.73	1.46	1.49	1.49	8.39	35.89	1.20
21	2.10	2.16	2.10	2.20	0.70	0.70	0.73	1.41	1.40	1.40	7.94	34.11	1.14
22	2.05	2.04	2.00	2.10	0.67	0.67	0.69	1.35	1.33	1.32	7.57	33.04	1.09
23	1.93	1.95	1.92	2.01	0.63	0.64	0.65	1.26	1.32	1.29	7.29	31.28	1.04
24	1.90	1.88	1.85	1.93	0.60	0.62	0.62	1.21	1.25	1.26	6.97	30.06	1.00
25	1.81	1.80	1.82	1.84	0.60	0.60	0.59	1.20	1.19	1.19	6.69	28.52	0.97
26	1.74	1.70	1.75	1.77	0.57	0.58	0.56	1.16	1.15	1.13	6.53	27.87	0.93
27	1.67	1.63	1.68	1.71	0.55	0.55	0.55	1.07	1.10	1.09	6.21	26.57	0.89
28	1.64	1.58	1.60	1.64	0.53	0.52	0.54	1.06	1.05	1.06	5.98	25.73	0.86
29	1.56	1.53	1.56	1.59	0.53	0.52	0.51	1.01	1.03	1.02	5.77	24.92	0.83
30	1.51	1.49	1.51	1.53	0.50	0.49	0.49	0.99	0.98	0.98	5.56	23.96	0.80
**Units**	Minutes	Minutes	Minutes	Minutes	Minutes	Minutes	Minutes	Minutes	Minutes	Minutes	Hours	Hours	Hours

Supplementary Table 1: Periods of oscillation for the first thirty modes of all data sets studied.

(15min,15min)	1-Week, 100 Miles<br>I-10 East	1-Week, ∼70 Miles<br>SoCal Network	1-Month, 400 Miles<br>I-5 North	Rain, 100 Miles<br>US-101 North	Avg
**MAE [Miles per Hour]**	1.57	1.88	1.7	1.84	1.74
**MRE**	.03	.03	.03	.037	.03
**RMSE**	3.26	3.41	3.36	3.63	3.41
**S/T-Corr.**	.92/.96	.89/.94	.89/.94	.90/.95	.9/.94

Supplementary Table 2: Summary of forecasting error analysis for the various examples studied.

Supplementary Note 1: Hankel Dynamic Mode Decomposition

Result: V, Λ, b_0

Inputs: Mean subtracted data matrix X, number of delays d

1. Embed mean subtracted data into H.

2. Form time shifted matrices H_1 and H_2.

3. Compute K

4. Compute eigendecomposition of K.

5. Compute the Koopman modes.

6. Compute initial amplitude coefficient.

Return: Koopman modes,eigenvalues and amplitude coefficient.

Supplementary References

1. Porter, G. Historic storm pounds southern california with damaging winds and record rain (2017). URL https://www.washingtonpost.com/news/capital-weather-gang/wp/2017/02/18/historic-storm-pounds-southern-california-with-high-winds-and-record-rain.

2. Tu, J. H., Rowley, C. W., Luchtenburg, D. M., Brunton, S. L. & Kutz, J. N. On dynamic mode decomposition: Theory and applications. *J. Comput. Dyn.* **1**, 391–421 (2014).

3. Mezić, I. & Banaszuk, A. Comparison of systems with complex behavior. *Physica D* **197**, 101 – 133 (2004).

4. Mezić, I. Spectral properties of dynamical systems, model reduction and decompositions. *Nonlinear Dyn.* **41**, 309–325 (2005).
