# Supplementary material for: Data-driven analysis and forecasting of highway traffic dynamics
Source: Nat Commun. 2020 Apr 29;11:2090. doi: 10.1038/s41467-020-15582-5 (PMC7190853; doi:10.1038/s41467-020-15582-5)
Supplement: Supplementary file 3 — Description of Additional Supplementary Files [file 41467_2020_15582_MOESM3_ESM.pdf]

Description of Additional Supplementary Files

File Name: Supplementary Movie 1 NGSIM US101 Multi-Lane Mode 1
Description: First Koopman mode for the NGSIM 101 multi-lane data

File Name: Supplementary Movie 2 NGSIM US101 Multi-Lane Mode 2
Description:Second Koopman mode for the NGSIM 101 multi-lane data

File Name: Supplementary Movie 3 NGSIM US101 Multi-Lane Mode 3
Description: Third Koopman mode for the NGSIM 101 multi-lane data

File Name: Supplementary Movie 4 NGSIM US101 Multi-Lane Mode 4
Description: Fourth Koopman mode for the NGSIM 101 multi-lane data

File Name: Supplementary Movie 5 NGSIM US101 Multi-Lane Mode 5
Description: Fifth Koopman mode for the NGSIM 101 multi-lane data

File Name: Supplementary Movie 6 NGSIM US101 Multi-Lane Mode 6
Description: Sixth Koopman mode for the NGSIM 101 multi-lane data

File Name: Supplementary Movie 7 NGSIM US101 Multi-Lane Mode 7
Description: Seventh Koopman mode for the NGSIM 101 multi-lane data

File Name: Supplementary Movie 8 NGSIM US101 Multi-Lane Mode 8
Description: Eighth Koopman mode for the NGSIM 101 multi-lane data

File Name: Supplementary Movie 9 NGSIM US101 Multi-Lane Mode 9
Description: Ninth Koopman mode for the NGSIM 101 multi-lane data

File Name: Supplementary Movie 10 NGSIM US101 Multi-Lane Mode 10
Description: Tenth Koopman mode for the NGSIM 101 multi-lane data

File Name: Supplementary Movie 11 NGSIM US101 Multi-Lane Mode 11
Description: Eleventh Koopman mode for the NGSIM 101 multi-lane data

File Name: Supplementary Movie 12 NGSIM US101 Multi-Lane Mode 12
Description: Twelfth Koopman mode for the NGSIM 101 multi-lane data

File Name: Supplementary Movie 13 NGSIM US101 Multi-Lane Mode 13
Description: Thirteenth Koopman mode for the NGSIM 101 multi-lane data

File Name: Supplementary Movie 14 NGSIM US101 Multi-Lane Mode 14
Description: Fourteenth Koopman mode for the NGSIM 101 multi-lane data

File Name: Supplementary Movie 15 LA Multilane Network Forecast
Description: Video of the forecasts for the Los Angeles multi-lane network data set
